# Seasonal Expression of Oxytocin and Oxytocin Receptor in the Scented Gland of Male Muskrat (*Ondatra zibethicus*)

**DOI:** 10.1038/s41598-017-16973-3

**Published:** 2017-11-30

**Authors:** Fengwei Zhang, Qian Liu, Ziyi Wang, Wenqian Xie, Xia Sheng, Haolin Zhang, Zhengrong Yuan, Yingying Han, Qiang Weng

**Affiliations:** 1 College of Biological Sciences and Technology, Beijing Forestry University, 100083 Beijing, China; 2 Department of Biosciences, University of Oslo, 0316 Oslo, Norway

## Abstract

Oxytocin (OT) can modulate multiple physiological functions via binding to the widely distributed oxytocin receptor (OTR). In this study, we investigated the seasonal expressions of OT, OTR and extracellular signal regulated kinase (ERK1/2) signaling pathway components in the scented gland of muskrat during the breeding and non-breeding seasons. Histologically, glandular cells, interstitial cells and excretory tubules were identified in the breeding season scented glands, whereas epithelial cells were sparse in the non-breeding season. Immunohistochemical results showed that OTR was present in epithelial cells and interstitial cells while OT, pERK1/2, ERK1/2 and c-fos were expressed in epithelial cells and glandular cells. The protein and mRNA expressions of OTR, OT and c-fos were significantly higher in the scented gland in the breeding season than in the non-breeding season. Importantly, the levels of OT in scented glands and serum were measured by hormone assays, and their concentrations were both significantly higher in the breeding season than in the non-breeding season. Moreover, bioinformatics analysis showed that the predicted targets of the differentially expressed microRNAs might include the genes encoding OTR, ERK1/2 and c-fos. These findings suggested that OT may regulate the function of muskrat scented glands by the locally expressed receptors.

## Introduction

Oxytocin (OT) is a nonapeptide hormone produced primarily in the neurons of the hypothalamic paraventricular nucleus and supraoptic nucleus and released into systemic circulation by posterior pituitary^1^. It is expressed as an inactive precursor, which goes through post-translational progressive hydrolysis facilitated by a series of enzymes before maturing into the active form^2^. OT plays an important role in lactation, parturition, maternal behavior and sexual reproduction in both sexes^3,4^. Meanwhile, OT is also synthesized in a variety of tissues besides the brain, including the corpus luteum^5^ and the placenta^6^ as well as testis^7^ and epididymis^8^.

The physiological functions of OT is mediated via binding to the widely distributed oxytocin receptor (OTR), which belongs to the rhodopsin-type (class I) G protein (Gαq11)-coupled receptors (GPCRs) family^9^. In humans, the *OXTR* gene is present in the genome as a single copy at the gene locus 3p25^10^. OTR is involved in the regulation of multiple physiological activities in peripheral tissues, such as the female uterine contractions and mammary gland milk ejection, as well as the male penile erection and ejaculation^10,11^. So far, studies about OTR function in peripheral organs have been mainly focusing on the reproductive system, with few reports on the non-reproductive organs, such as the scented gland.

Upon activation, OTR initiates different intracellular signaling pathways, many of which have not been studied in depth. Among them, the extracellular signal–regulated kinase 1/2 (ERK1/2) is one of the most important pathways^12–14^. Activated ERK1/2 plays a critical role in delivering the extracellular stimuli from the surface receptor to the nucleus, which then triggers context-dependent biological effects, such as cell proliferation, differentiation, morphology maintenance, cytoskeleton construction and apoptosis^15,16^. Phosphorylated ERK1/2 (pERK1/2) translocates from the cytoplasm to the nucleus, which in turn activates multiple transcription factors. For example, nuclear pERK1/2 mediates proliferative effects via mechanisms that trigger the induction of c-fos expression^14,17,18^. In the rat myometrium, ERK1/2 activation has been shown to be important for the OTR-mediated myometrial contraction and labor^12,19^.

miRNAs, a class of non-coding single-stranded RNA molecules (about 18–24 nucleotides), are involved in post-transcriptional gene expression regulation by inhibiting mRNA translation with or without transcript degradation^20^. Numerous studies have shown that miRNAs participate in a series of important biological processes, including early development, cell proliferation and differentiation, metabolism and tissue morphogenesis^21,22^. However, the expression and function of most miRNAs have not been fully characterized, especially in wild seasonal breeding animals.

The muskrat (*Ondatra zibethicus*) is a medium-sized, semi-aquatic rodent native to North America and later introduced into China^23,24^. The muskrat is a typical long-day wild seasonal breeder whose annual life cycle can be roughly divided into the breeding season (March to October) and the non-breeding season (November to next February). Notably, the male muskrat has a pair of scented glands located between the skin and muscle at the ventral base of the tail. During the breeding season, in order to attract females, the scented gland will significantly expand and secrete musk (perfume substances), which is also a common and costly ingredient in traditional Chinese medicine^23,25^.

In addition to the central nervous system, OT can also be produced in and act on the peripheral system, such as exocrine glands. Yet, whether there is a role of OT in the scented gland of muskrat remains unclear. In this study, we investigated the expression of OT and OTR in the scented gland of male muskrats during the breeding and non-breeding seasons. Furthermore, as a readout of the OTR signaling output, we also evaluated the phosphorylation of ERK1/2 and the expression of c-fos in the scented gland. Meanwhile, the different expression of miRNAs in the scented gland during different seasons were measured and their targeting genes were predicted.

## Results

### Morphological and histological features of the muskrat scented gland

**Figure 1:**
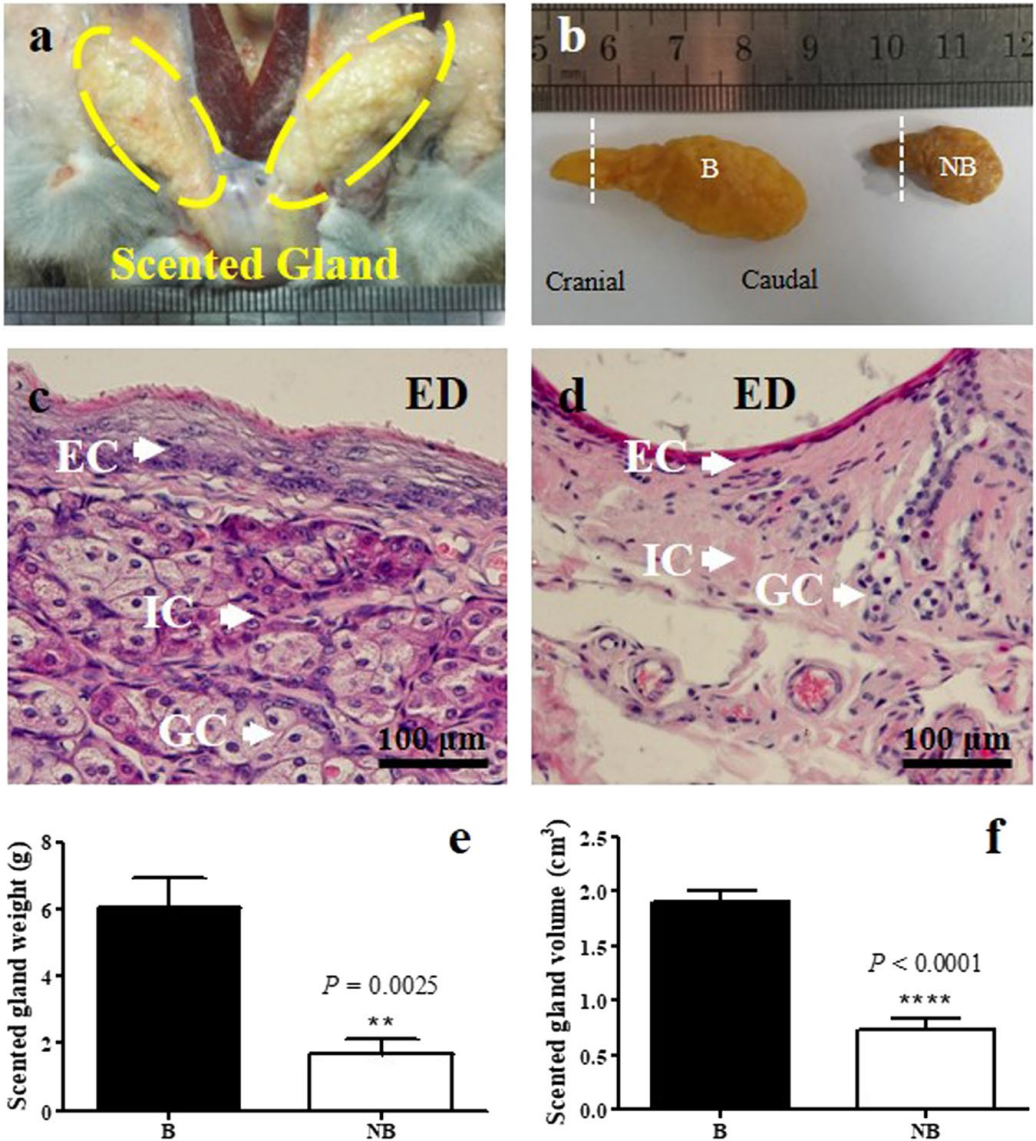
Morphological and histological features of the scented gland of muskrats in the breeding season and the non-breeding season. (**a**) Anatomic localization and morphology of the scented gland. (**b**) Morphological features (including cranial and caudal indicators) of scented glands after fixation; the dotted line indicates the location of the tissue slice. Histological observation of the scented gland in the breeding season (**c**) and the non-breeding season (**d**). (**e**) The average weight of scented glands in breeding and non-breeding seasons. (**f**) The average volume of scented glands in breeding and non-breeding seasons. Scale bars = 100 μm. B, breeding season; NB, non-breeding season; GC, glandular cell; EC, epithelial cell; IC, interstitial cell; ED, excretory duct. The error bars represent means ± s.e.m. for five independent experiments. ^**^ *P < *0.01; ^****^ *P* < 0.0001.

Morphological observation of the scented gland during breeding and non-breeding seasons was performed (Fig. 1). Scented glands were located between the skin and muscle at the ventral base of the tail (Fig. 1a). The post-fixed scented gland showed morphological changes in the breeding season versus the non-breeding season (Fig. 1b). In addition, histological observation revealed three types of cells, including epithelial cells, glandular cells and interstitial cells (Fig. 1c,d), which was in accordance with our previous studies^26,27^. The average weight and volume of scented gland in the breeding season were significantly higher than those in the non-breeding season (Fig. 1e,f).

### Immunohistochemical localization of OTR, OT, pERK1/2, ERK1/2, c-fos in the scented gland of muskrats

**Figure 2:**
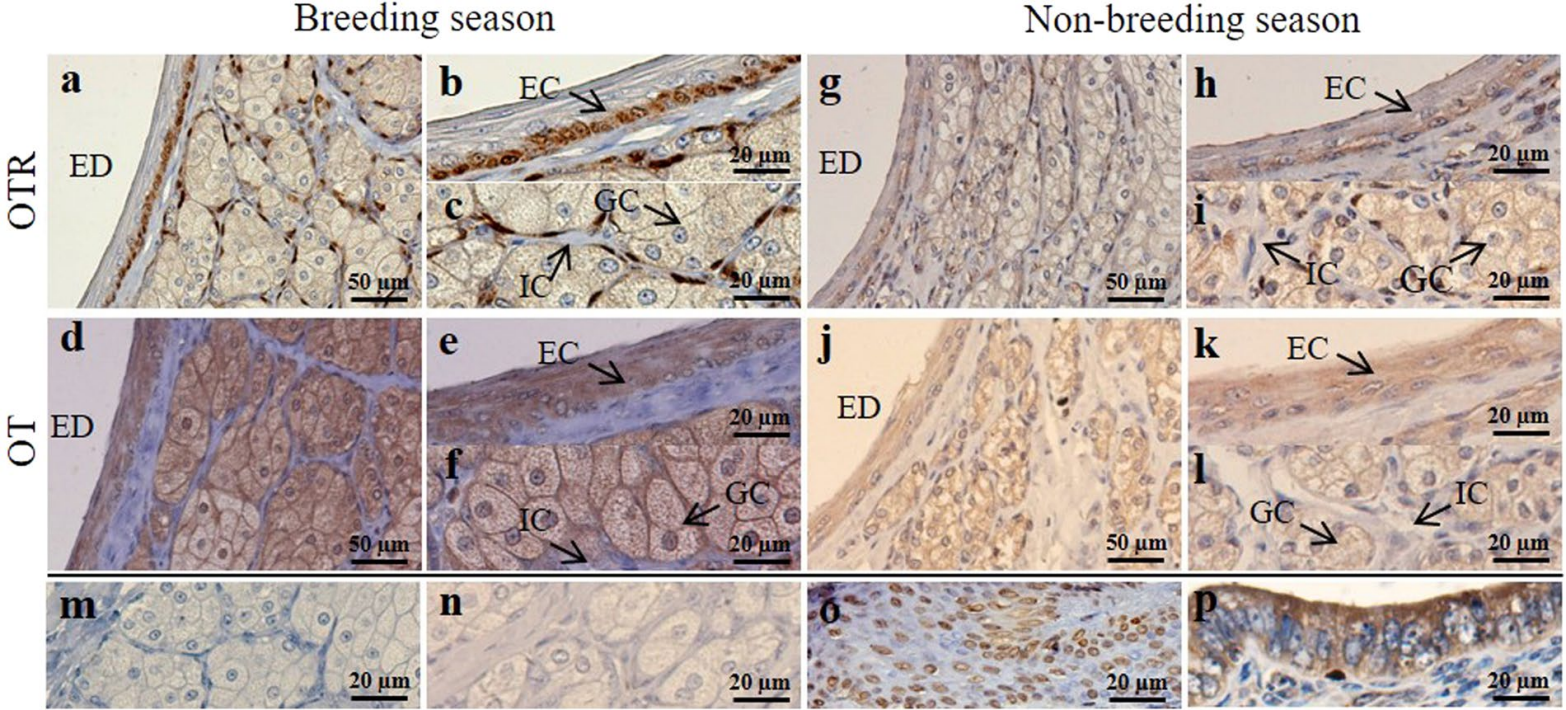
Seasonal immunolocalization of OTR and OT in the scented gland of muskrat. Black arrows indicate the three cell types: glandular cells, epithelial cells and interstitial cells. The first (**a**,**d**) and second (**b**,**c**,**e**,**f**) columns represent staining in the breeding season. The third (**g**,**j**) and fourth (**h**,**i**,**k**,**l**) columns represent staining in the non-breeding season. (**a**–**c**, **g**–**i**) Immunolocalization of OTR in scented glands. (**d**–**f**, **j**–**l**) Immunolocalization of OT in scented glands. (**m**,**n**) Negative control, sections were treated with normal rabbit serum instead of primary antisera, represent the breeding season and non-breeding season, respectively. (**o**,**p**) The mammary gland and uterus of female muskrats were used as positive control for OTR and OT, respectively. GC, glandular cell; EC, epithelial cell; IC, interstitial cell; ED, excretory duct. Scale bars = 50 µm (**a**,**d**,**g**,**j**); 20 µm (**b**,**c**,**e**,**f**,**h**,**i**,**k**,**l**,**m**,**n**,**o**,**p**).

The seasonal immunohistochemical localizations of OTR and OT in the scented gland of muskrats are shown in Fig. 2. In the breeding season, strong immunoreactivity of OTR was present in the epithelial cells (Fig. 2b) and interstitial cells (Fig. 2c), but not in glandular cells (Fig. 2c). In contrast, the immunoreactivity of OTR was much weaker in both epithelial cells (Fig. 2h) and interstitial cells (Fig. 2i) in the non-breeding season. The expression of OTR and OT in mammary gland (Fig. 2o) and uterus (Fig. 2p) of female muskrats were used as positive controls.

The immunoreactivity of OT was detected in the cytoplasm of epithelial cells (Fig. 2e) and glandular cells (Fig. 2f), but not in interstitial cells (Fig. 2f), in both seasons. Compared with the breeding season, the OT immunostaining intensity was weaker in the non-breeding season (Fig. 2j–l). No signal was observed in the negative controls (Fig. 2m,n).

**Figure 3:**
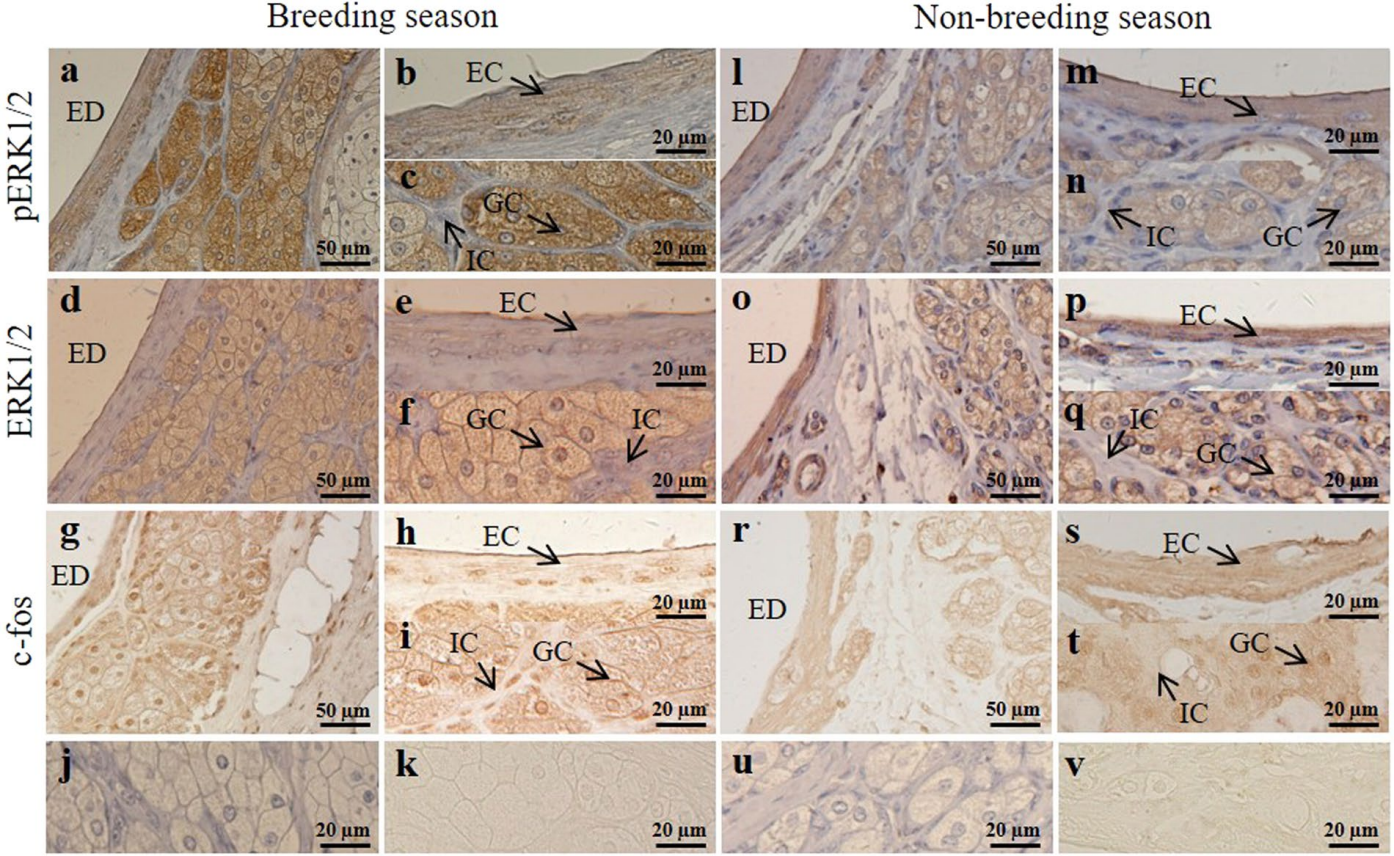
Seasonal immunolocalization of pERK1/2, ERK1/2 and c-fos in the scented gland of muskrats. Black arrows indicate three types of cells including glandular cells, epithelial cells and interstitial cells. The first (**a**,**d**,**g**,**j**) and second (**b**,**c**,**e**,**f**,**h**,**i**,**k**) column represents staining in the breeding season. The third (**l**,**o**,**r**,**u**) and fourth (**m**,**n**,**p**,**q**,**s**,**t**,**v**) column represents staining in the non-breeding season. (**a**–**c**, **l**–**n**) Immunolocalization of pERK1/2 in scented glands. (**d**–**f**, **o**–**q**) Immunolocalization of ERK1/2 in scented glands. (**g**–**i**, **r**–**t**) Immunolocalization of c-fos in scented glands (without haematoxylin staining). (**j**,**k**,**u**,**v**) Negative control, sections were treated with normal rabbit serum instead of primary antisera. (**k**,**v**) Negative control without haematoxylin staining, GC, glandular cell; EC, epithelial cell; IC, interstitial cell; ED, excretory duct. Scale bars = 50 µm (**a**,**d**,**g**,**l**,**o**,**r**); 20 µm (**b**,**c**,**e**,**f**,**h**,**i**,**j**,**k**,**m**,**n**,**p**,**q**,**s**,**t**,**u**,**v**).

The immunostaining signals of pERK1/2 and ERK1/2 were observed in the epithelial cells (Fig. 3b,e,m and p) and glandular cells (Fig. 3c,f,n and q), but not in interstitial cells (Fig. 3c,f,n and q). Specifically, the immunoreactivity of pERK1/2 and ERK1/2 in epithelial cells were at the same level, while in glandular cells, the immunostaining in the breeding season were more intense than in the non-breeding season. Interestingly, c-fos was observed in the nuclei of all three cell types in the breeding season (Fig. 3h,i), but only in glandular cells nuclei in the non-breeding season (Fig. 3t). No signal was observed in the negative controls (Fig. 3j,k,u and v). All staining images were quantified and summarized in Table 1.

**Table 1:** Immunohistochemical localization of OT, OTR, ERK1/2, pERK1/2 and c-fos in the scented gland of muskrats.

	EC		GC		IC	
	B	NB	B	NB	B	NB
OTR	+++	+	−	−	+++	+
OT	++	+	++	+	−	−
pERK1/2	+	+	+++	+	−	−
ERK1/2	+	+	++	+	−	−
c-fos	++	−	++	+	+	−

EC, epithelial cells; GC, glandular cell; IC, interstitial cells. B, breeding season: NB, non-breeding season. −, negative staining; +, positive staining; ++ , strong positive staining; +++ , very strong positive staining.

### The protein expressions of OTR, pERK1/2, ERK1/2, c-fos in the scented gland of muskrats

**Figure 4:**
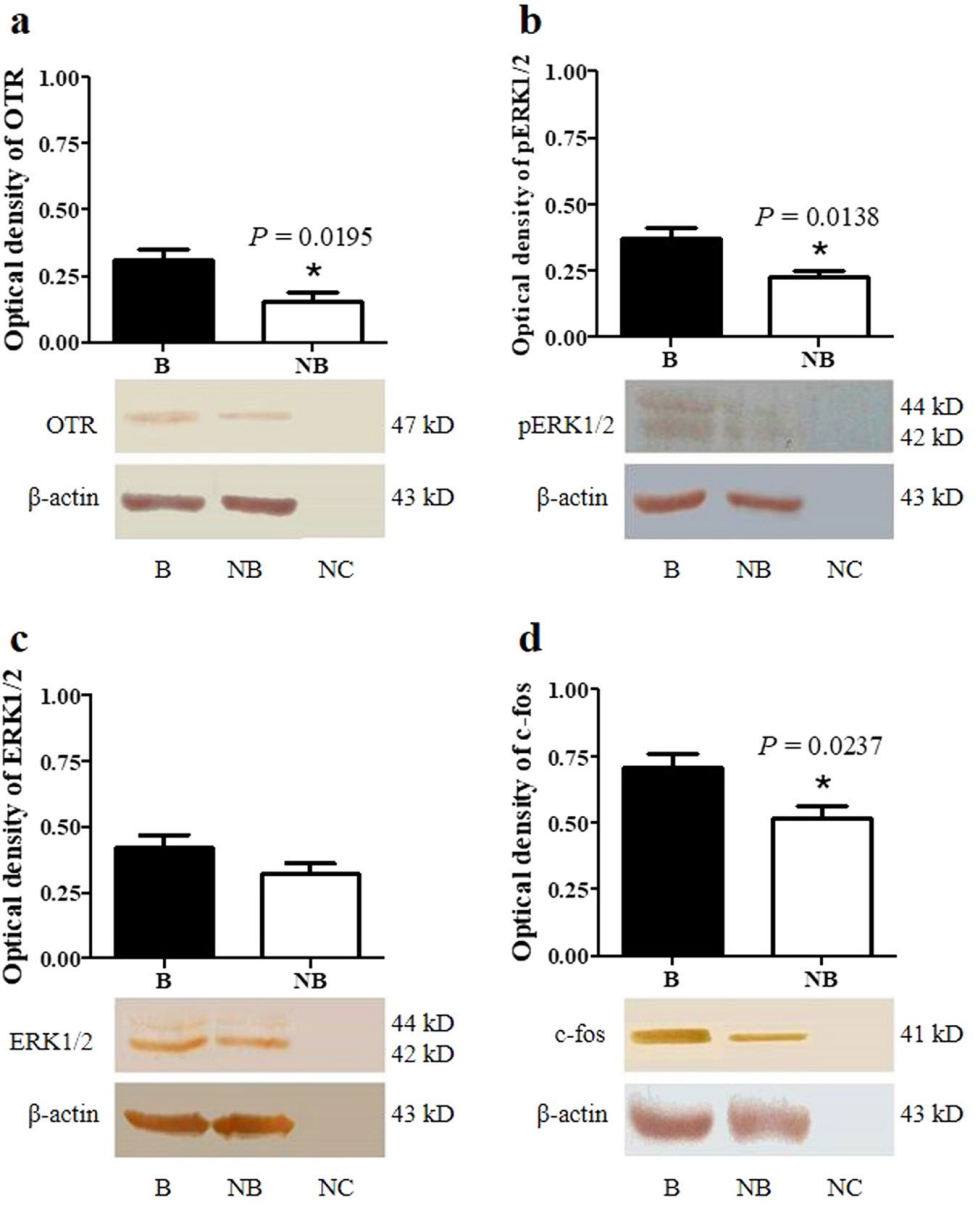
Seasonal protein expression of OTR (**a**), pERK1/2 (**b**), ERK1/2 (**c**) and c-fos (**d**) in the scented glands. The error bars represent means ± s.e.m. for five independent experiments. B, breeding season; NB, non-breeding season; NC, negative control. ^*^ *P* < 0.05.

The seasonal protien expression of OTR, pERK1/2, ERK1/2 and c-fos in the muskrat scented gland was examined by Western analysis. Antibody pre-absorption was also performed as a negative control (Fig. 4 lane NC). The major bands for OTR, ERK1/2 (including pERK1/2) and c-fos were detected at a molecular weight of about 47 kDa, 44/42 kDa and 41 kDa, respectively. The protein levels of OTR, pERK1/2 and c-fos in scented glands were significantly higher in the breeding season than those in the non-breeding season.

### The mRNA expressions of Oxt, Oxtr, Mapk3, Mapk1, Fos in the scented gland of muskrats

**Figure 5:**
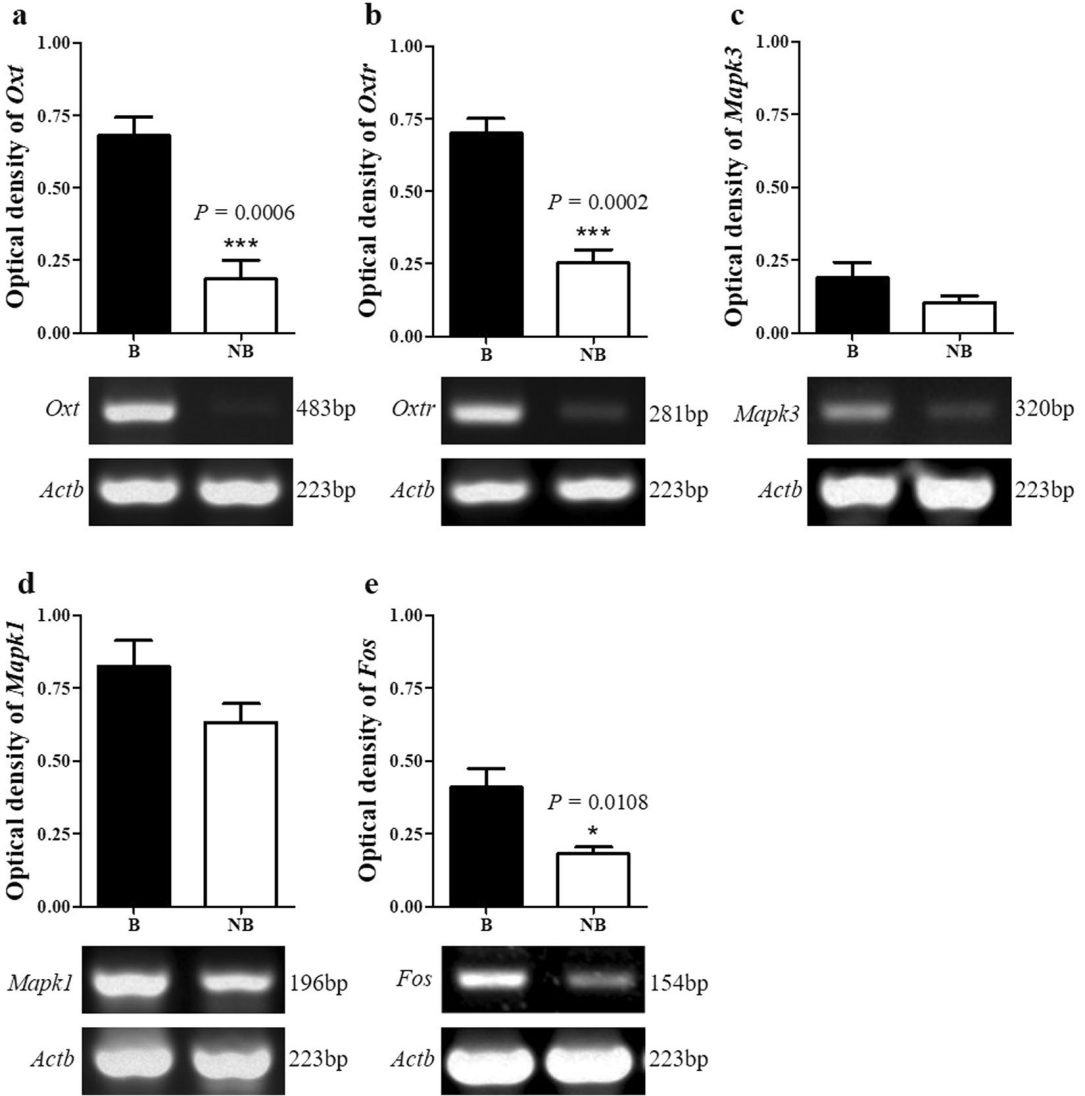
Seasonal mRNA expression of *Oxt* (**a**)*, Oxtr* (**b**)*, Mapk3* (**c**)*, Mapk1* (**d**) and *Fos* (**e**) in the scented glands. The error bars represent means ± s.e.m. for five independent experiments. B, breeding season; NB, non-breeding season. ^*^ *P* < 0.05; ^***^ *P < *0.001.

The mRNA levels of *Oxt, Oxtr, Mapk3, Mapk1, Fos* (genes encoding OT, OTR, ERK1/2, c-fos respectively) were assessed by RT-PCR (Fig. 5). Consistenly, the mRNA levels of *Oxt, Oxtr, Fos* were significantly higher in the breeding season than those in the non-breeding season (Fig. 5a,b and e), while the expressions of *Mapk3/Mapk1* mRNA showed no difference between the two different seasons (Fig. 5c,d).

### Bioinformatics analysis of miRNAs in the scented gland of muskrats

The seasonal expression of global miRNAs in this tissue was reported previously^23^. To search for the miRNAs that may have a direct impact on the OT-OTR-ERK signaling, we used two different software packages, miRanda and TargetScanHuman, to predict their target genes based on the sequences of these miRNAs and the known mRNAs. Notably, *Oxtr* was predicted as the target genes of hsa-miR-202-5p, mmu-miR-126b-5p and mmu-miR-5119 by TargetScanHuman software, while mmu-miR-126b-5p and mmu-miR-5119 were also predicted to target *Mapk1*. Meanwhile, hsa-miR-762 was predicted to target *Mapk3* and *Mapk1* by both software packages, and *Fos* was predicted as a target of hsa-miR-4454 and rno-miR-144-3p by TargetScanHuman software. The differential expression summary of these selected miRNAs is shown in Table 2.

**Table 2:** The differential expression of 16 miRNAs in the scented gland in the breeding season compared with the non-breeding season.

miRNAs	Target genes	Expression differences
mmu-miR-126b-5p	*Oxtr* ^b^, *Mapk1* ^b^	Up
hsa-miR-1	*Mapk3* ^a^, *Mapk1* ^a^	Up
mmu-miR-7058-3p	*Mapk1* ^b^	Up
hsa-miR-202-5p	*Oxtr* ^b^	Up
mmu-miR-1b-5p	*Mapk1* ^b^	Up
hsa-miR-762	*Mapk3* ^a,b^, *Mapk1* ^a,b^	Up
rno-miR-144-3p	*Fos* ^b^	Up
mmu-miR-5119	*Oxtr* ^b^, *Mapk1* ^b^	Up
hsa-miR-4485	—	Up
rno-miR-144-5p	—	Up
rno-miR-451-5p	—	Up
hsa-miR-4454	*Fos* ^b^	Down
hsa-miR-5100	*Mapk1* ^b^	Down
mmu-miR-8112	—	Down
mmu-miR-6937-5p	—	Down
rno-miR-147	—	Down

“—”, no more target genes; “a”, the miRNA targets predicted by miRanda; “b”, the miRNA targets predicted by TargetScanHuman.

### The concentration of OT in scented glands and plasma

**Figure 6:**
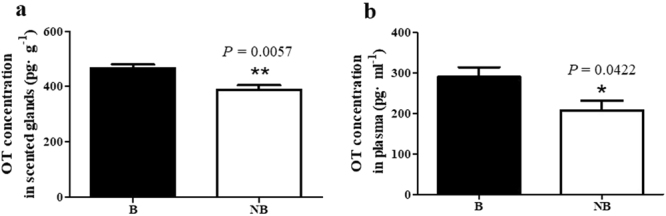
Seasonal OT concentrations in scented glands (**a**) and plasma (**b**). The error bars represent means ± s.e.m. for five independent experiments. B, breeding season; NB, non-breeding season. ^*^ *P* < 0.05; ^**^ *P < *0.01.

The seasonal concentrations of OT in scented glands and plasma were analyzed (Fig. 6). In the scented glands, the OT level was significantly lower in the non-breeding season (389 ± 35 pg∙g^−1^) as compared to the breeding season (466 ± 30 pg∙g^−1^) (Fig. 6a). Similar trend was also observed in the plasma, as the OT level decreased from 289 ± 54 pg∙ml^−1^ in the breeding season to 207 ± 53 pg∙ml^−1^ in the non-breeding season (Fig. 6b).

## Discussion

In the present study, we have used different approaches to investigate the seasonal expression profiles of OT, OTR and ERK1/2 signaling pathway components in the scented gland of male muskrat. To our knowledge, this is the first report showing the expression of both OT and OTR in the scented gland. These findings suggested that the scented gland may be able to synthesize OT, which is likely to play important regulatory roles via OTR in scented glands.

As a male-specific organ, the weight and volume of the scented gland in the breeding season were significantly higher than in the non-breeding season, indicating that its function may be associated with the reproductive status of male muskrats. Our previous studies showed that the plasma concentrations of follicle stimulating hormone, luteinizing hormone and prolactin in the male muskrat were higher in the breeding season than in the non-breeding season^23,28^. Similarly, OT, another key hormone released by the pituitary, also showed a similar seasonal expression profile. Consistent with the hormone profile, the mRNA and protein expression of OT was higher in the breeding season than in the non-breeding season as well. Furthermore, the immunoreactivity of OT was mainly located in the cytoplasm of glandular cells and epithelial cells, and a stronger staining was observed in the breeding season. Previously, the local production of OT in peripheral organs has been reported, especially in the male reproductive tract. The synthesis of OT in the testis and epididymis has been demonstrated in many animals, including rat, sheep and human^29^. In guinea pigs, *in vitro* cultured Leydig cells were able to synthesize OT *de novo* ^30^. In ovines, the level of OT produced in the testis and epididymis increased during puberty^31^. In brushtail possums, the seasonal changes of local mesotocin (an OT-like peptide in marsupials) concentration may be related to the growth and regression of their prostate^32^. Similarly, our results suggest that OT may be synthesized in the glandular cells and epithelial cells of scented glands and its level closely associated with the seasonal changes of scented glands morphology. In addition, the muskrat scented gland function may be regulated by the OT from both the hypothalamus-pituitary system (endocrine manner) and the scented gland itself (autocrine/paracrine manner).

In males, OT plays an essential role in multiple physiological functions, such as the contractility of the reproductive tract. In the testis and epididymis, OT promotes sperm transport and maturation by increasing tubules contraction. In the rat testis, for example, lack of Leydig cells showed an extremely low level of OT and significantly weakened seminiferous tubule activity *in vitro* ^33^. Meanwhile, the number of spermatozoa in the sheep’s cauda epididymis was significantly increased after 10 min of OT treatment^34^. In addition, OT may also regulate penile contraction and erection. Studies have showed that the OT concentration in the corpus cavernosum blood increased during penile erection^29^, and the immunolocalization of OTR in the endothelial and smooth muscle of the rabbit and human corpus cavernosum has also been reported^35^. In this study, the expression of OTR in the scented gland suggested that the scented gland of male muskrats may be a target organ of OT. Immunohistochemical observation clearly showed that OTR immunoreactivity was mainly distributed in the cell membrane of epithelial cells and interstitial cells, but not in that of glandular cells. The mRNA and protein expression of OTR further confirmed its presence in the scented gland. Importantly, both levels were significantly higher in the breeding season when the scented gland secreted musk. Taken together published and current results, we speculated that OT may regulate the contraction of scented glands in the breeding season to facilitate musk secretion.

It is well established that OTR can activate the ERK1/2 signaling pathway in different cell types. In human myometrial cells, endogenous OTR interacts with β_2_-adrenergic receptor activated ERK1/2 to modulate a variety of uterine activities, such as contraction and labor^12^. In mice, long-term OT treatment exacerbated prostate hyperplasia by activating the ERK1/2 pathway^36^. In this study, we examined the seasonal expression of phospho- and total ERK1/2 in the scented gland. Immunohistochemical observation showed that pERK1/2 and ERK1/2 were localized in epithelial cells and glandular cells of the scented gland. Western analysis revealed that pERK1/2 level was significantly higher in the breeding period compared to the non-breeding season, with no significant difference in the expression of ERK1/2. Previous studies have shown that the activated ERK1/2 signaling pathway located in the cytoplasm may induce the contraction in myometrial cells^14,37^. Therefore, our results implied that OT/OTR may be involved in the activation of the ERK1/2 signaling pathway, consequently affecting the glandular contraction and musk secretion. In addition, nuclear ERK1/2 was considered to regulate cell proliferation by inducing c-fos expression^13,14^. Our previous study demonstrated that in the breeding season, the number of epithelial cells and glandular cells in the scented gland markedly increased, suggesting a seasonal change in cell proliferation^23,26,27,38,39^. The present study showed a stronger immunostaining of c-fos in the epithelial cells and glandular cells as well as higher c-fos mRNA and protein expression in the breeding season relative to the non-breeding season. These results suggested that the hyperactive ERK signaling and high level of c-fos in the breeding season may promote the proliferation of epithelial cells and glandular cells.

miRNAs, a type of small non-coding RNAs, play critical roles in regulating the gene expression outcome. 16 differentially expressed miRNAs have been identified in the scented glands of muskrats during the seasonal cycle. Among those, 10 were predicted to target *Oxtr, Mapk3, Mapk1* and *Fos* by two different algorithms, implicating that OTR and the activated ERK1/2 pathway may be important for the scented gland function. Notably, the differential expression of miRNA hsa-miR-4454, predicted to target *Fos*, was consistent with changes in the *Fos* mRNA level, suggesting that hsa-miR-4454 may be involved in regulating the *Fos* expression in the scented glands. However, the expression of hsa-miR-202-5p, mmu-miR-126b-5p and mmu-miR-5119, all of which target *Oxtr*, were contradictory to the change in the *Oxtr* mRNA expression. The reason for these inconsistencies is unknown at present and needs further characterization. Determining their roles in this seasonal reproduction process as well as correlation with the expression of their target genes is currently ongoing. Overall, although these results were insufficient to elucidate the relationship between these miRNAs and scented glands function, they provided important clues for the functional analysis of miRNA in muskrats for future studies.

In conclusion, our results described the seasonal expression and localization of OT and OTR in the scented gland of male muskrats. These findings suggested that the regulation of OT in the scented gland may be mediated via endocrine, autocrine or paracrine mechanisms; OTR may be required for the contraction of the scented gland to promote musk secretion during the breeding season. Furthermore, the matching expression profiles of phospho- and total ERK1/2 as well as c-fos implied that OT/OTR may regulate the function of scented glands through ERK1/2 signaling, in which may subsequently up-regulate c-fos expression to promote the scented gland expansion in the breeding season. This study provides new insight into the function of OT in scented glands, and lays the foundation for future mechanistic studies.

## Methods

### Animals and tissues collection

Twenty adult muskrats were obtained in the breeding season (n = 10) and the non-breeding season (n = 10) from Jinmu Muskrats Breeding Farm, Hebei Province, China. The muskrats were kept with a pattern of one male and one female in one enclosure. All the procedures on animals were carried out in accordance with the Policy on the Care and Use of Animals by the Ethical Committee, Beijing Forestry University and approved by the Department of Agriculture of Hebei province, PR China (JNZF11/2007). Male muskrats were weighed and deeply anesthetized with ether and then a pair of scented glandular tissues was obtained from the male muskrat. One of the scented glands was immediately fixed overnight in Bouin’s solution and then stored in 70% ethanol for histological and immunohistochemical observations; the other was immediately stored at −80 °C until used for Western blotting, RT-PCR and hormone assays. Plasma samples were immediately collected and stored at −20 °C for hormone assays. The samples were collected from 2013 to 2016 and the analysis was repeated by different experimenters.

### Histology

Scented glands were dehydrated in ethanol series and embedded in paraffin. Serial sections (5 μm) were mounted on slides coated with poly-L-lysine. Some sections were stained with hematoxylin-eosin (HE) for observations of general histology. The rest of the sections were processed for immunohistochemistry.

### Immunohistochemistry

Briefly, serial sections were incubated with 10% normal goat serum to reduce background staining and then incubated with primary rabbit polyclonal antibodies (Bioss Antibodies, Beijing, China, 1:200 dilutions) against OTR (bs-1314R)^40,41^, OT (bs-17582R), ERK1/2 (bs-2637R), phospho-ERK1/2 (S202 + Y204, bs-3016R), or c-fos (bs-10172R) for 12 h under 4 °C. The sections were then incubated with a secondary antibody, goat anti-rabbit lgG conjugated with biotin and peroxidase with avidin, using rabbit ExtrAvidin^TM^ Peroxidase staining kit (Sigma Chemical Co., St. Louis, MO, USA) was performed, followed by visualizing with 20 mg 3, 3-diaminobenzidine (Wako, Tokyo, Japan) solution in 100 ml of 0.05 M Tris–HCl buffer, pH 7.6, plus 20 μl H_2_O_2_. The control sections were treated with normal rabbit IgG (c-0006, Bioss Antibodies) at 1:20 dilutions instead of the primary antibody. A semi-quantitative score for positivity (− negative, to +++ very strong positive) was used by two independent experimenters blinded to the animal group^42,43^.

### Western blotting

Scented glands were weighed and diced into small pieces using a clean razor blade. Tissues were homogenized in a homogenizing buffer for 30 min on ice. Homogenates were centrifuged at 12,000 *g* for 6 min at 4 °C. Protein extracts (25 μg) were mixed with an equal volume of 2 × Laemmli sample buffer. Equal amounts of each sample were loaded and run on a 10% SDS-PAGE gel at 18 V∙cm^−1^ and transferred to nitrocellulose membranes using a wet trans-blotting apparatus (Bio-Rad, Richmond, CA, USA). The membranes were blocked in 3% BSA for 1 h at room temperature. Primary incubation of the membranes used a 1:500 dilution of rabbit anti-rat OTR, ERK1/2, phospho-ERK1/2, or c-fos antibody for overnight at 4 °C. Secondary incubation of the membrane used a 1:1000 dilution of goat anti-rabbit IgG tagged with horseradish peroxidase for 60 min at room temperature. Finally, the membrane was colored with 10 mg 3, 3-diaminobenzidine solution in 50 ml phosphate buffer (0.03 M) plus 3 μl H_2_O_2_. β-actin was selected as the endogenous control. Antibody pre-absorptions were performed here as a negative control. The antigen used for pre-absorption is: OTR (bs-1314P, Bioss Antibodies); ERK1/2 (1240 S, Cell Signaling Technology, Danvers, MA, USA); phospho-ERK1/2 (S202 + Y204) (1150 S, Cell Signaling Technology); c-fos (bs-0469P, Bioss Antibodies); β-actin (1025, Cell Signaling Technology).

### RNA isolation

Total RNAs from each sample were extracted using TRIzol Reagent (Invitrogen Co., CA, USA) according to the manufacturer’s protocol. Approximately 100 mg of scented glands were thawed and immediately homogenized in 1 ml of TRIzol Reagent by ultrasonic crusher. The homogenate was incubated for 15 min at room temperature to ensure the complete dissociation of nucleoprotein complexes. After the addition of 0.2 ml of chloroform, the mixture was vigorously shaken for 15 sec at room temperature and centrifuged at 12,000 *g* for 15 min at 4 °C. The aqueous phase was then transferred to a fresh tube and an equal volume of isopropanol was added. Then the sample was kept for 10 min at room temperature. RNA was precipitated by centrifugation at 12,000 *g* for 15 min at 4 °C. The RNA pellet was washed twice with 70% ethanol, briefly dried under air, and dissolved in 50 µl of diethylprocarbonate-treated water.

### RT-PCR

The first-strand cDNA from total RNA was synthesized using StarScript II First-strand cDNA Synthesis Mix (GenStar, Beijing, China). The 20 μl of reaction mixture contained 3 μg of total RNA, 1 μl of Oligo (dT)_18_, 1 μl of StarScript II RT Mix, 10 μl of 2 × Reaction mix, 5 μl of diethylprocarbonate-ddH_2_O. The PCR amplification was performed with 20 µl of reaction mixture containing 1 µl of first-strand cDNA, 1 µl each primer (10 µM), 7 µl ddH_2_O, 10 µl 2 × *Taq* PCR StarMix with Loading Dye (GenStar, Beijing, China) under the following condition: 94 °C for 2 min for the initial denaturation of the RNA/cDNA hybrid, 35 cycles of 94 °C for 30 sec, 58 °C for 30 sec and 72 °C for 30 sec with a final extension of 5 min at 72 °C. The first-strand cDNA was used for PCR amplification with the following primers (Table 3). The PCR product was electrophoresed in the 1% agarose gel for 30 min and individual bands were visualized by GelRed (Biotium Inc., Fremont, CA, USA) staining. The housekeeping gene *Actb* was selected as the endogenous control. The bands were quantified using Quantity One software (Version 4.5, Bio-Rad, Richmond, CA, USA) and the related expressions relative to *Actb* were calculated. After obtaining the sequence of each PCR product, we blasted with the known mRNA sequences of rat, mouse, bovine and human, found the homologous sequence fragment in each species and compared for homology (Table 4).

**Table 3:** Oligonucleotide primers used for RT-PCR.

Gene Name	Primers	Product size (bp)
*Oxt*	F: CTTGGCCTACTGGCTCTGAC	483
	R: GGGCAGGTAGTTCTCCTCCT	
*Oxtr*	F: CCTACGTCACATGGATCACG	281
	R: CCACATCTGCACGAAGAAGA	
*Mapk3*	F: CTGAGCAACGACCACATCTG	320
	R: TCATGTTCAGGGTCAGCGAT	
*Mapk1*	F: TACCTTGACCAGCTGAACCA	196
	R: AGCTTTGGAGTCAGCGTTTG	
*Fos*	F: GAGGGGCAAGGTAGAACAGT	154
	R: GGTTGGCAATCTCAGTCTGC	
*Actb*	F: GACTCGTCGTACTCCTGCTT	223
	R: AAGACCTCTATGCCAACACC

**Table 4:** Similarity between muskrat, rat, mouse, bovine, and human genes.

Gene name	Rat (%)	Mice (%)	Bovine (%)	Human (%)
*Oxt*	85.93	86.45	87.39	89.63
*Oxtr*	92.37	92.16	95.21	86.10
*Mapk3*	93.33	94.07	91.11	93.18
*Mapk1*	87.21	84.19	85.38	83.11
*Fos*	90.15	86.67	84.28	82.93
*Actb*	90.01	91.16	83.62	88.37

### MicroRNAs-sequencing and bioinformatics analysis

The miRNA sequencing and analysis were previously described in detail^23,44^. Briefly, the small RNA (sRNA) libraries for the scented gland of muskrat from the breeding season (named: SGB1) and the non-breeding season (named: SGNB2) were constructed from total RNAs using the Illumina Truseq Small RNA Preparation kit (RS-930-1012, Illumina Inc., USA), and were sequenced on the Illumina GAIIx platform following the vendor’s recommended protocol at Beijing Yuanquanyike Biological Technology Co., Ltd (Beijing, China). A proprietary pipeline script, ACGT101-miR v4.2 (LC Sciences, Houston, TX, USA), was utilized to analyze the sequencing data. The sRNAs were annotated by comparison with the deposited sequences in the NCBI GenBank (http://www.ncbi.nlm.nih.gov/) and the Rfam11.0 databases (http://rfam.sanger.ac.uk/). The remaining sequences were used to BLAST search against miRBase (version 20, http://www.mirbase.org/) to identify known miRNAs. Potential novel miRNAs candidates were predicted by Mireap (version 0.2, http://sourceforge.net/projects/mireap/). Possible target genes regulated by miRNAs were predicted using the miRanda (version 3.3a, http://www.microrna.org/microrna/) and Target Scan Human (version 7.0, http://www.targetscan.org/vert_70/). R software was utilized to analyze the correlation between differential expression profile of miRNAs and their targeted genes.

### Hormone Assays

The scented gland and plasma samples from each animal were analyzed by the enzyme linked immunosorbent assay (ELISA) to detect OT concentrations using the ELISA Kit (Kit CSB-E14197r for OT, Cusabio Biotech Co., Ltd., Wuhan, China). Samples preparation followed the user manual. Tissue samples were rinsed with PBS, and then homogenized in 1 ml of PBS and stored overnight at −20 °C. After two freeze-thaw cycles were performed to break the cell membranes, the homogenates were centrifuged for 5 min at 5000 g at 4 °C. Then the supernatant was collected and assayed. Plasma samples were centrifuged for 15 min at 3000 *g* at 4 °C, and then the supernatant was collected and assayed. The minimum level of OT detection of this ELISA kit is 9.375 pg∙ml^−1^. The intra/inter-assay variation were both less than 15% for OT.

### Statistical analysis

Statistical comparisons were made with the Student’s t-test using Prism 5 (Graphpad Software Inc., CA, USA). *P* < 0.05 was considered statistically significant.

### Data Availability

The datasets generated during and analysed during the current study are available from the corresponding author on reasonable request.

## Electronic supplementary material

Supplementary Information
