# Supplementary material for: Seasonal Expression of Oxytocin and Oxytocin Receptor in the Scented Gland of Male Muskrat (Ondatra zibethicus)
Source: Sci Rep. 2017 Nov 30;7:16627. doi: 10.1038/s41598-017-16973-3 (PMC5709462; doi:10.1038/s41598-017-16973-3)
Supplement: Supplementary file 1 — Supplementary Information [file 41598_2017_16973_MOESM1_ESM.pdf]

Seasonal Expression of Oxytocin and Oxytocin Receptor in the Scented Gland of Male Muskrat (*Ondatra zibethicus*)

Fengwei Zhang[1], Qian Liu[1], Ziyi Wang[1], Wenqian Xie[1], Xia Sheng[2], Haolin Zhang[1], Zhengrong Yuan[1], Yingying Han[1*], Qiang Weng[1*].

[1]*College of Biological Sciences and Technology, Beijing Forestry University, 100083 Beijing, China.*

[2]*Department of Biosciences, University of Oslo, 0316 Oslo, Norway.*

[*]Correspondence:

Yingying Han, Ph.D., Laboratory of Animal Physiology, College of Biological Sciences and Technology, Beijing Forestry University, Beijing 100083, China. Fax: +86 10-6233-6399; E-mail: thinkinghyy@bjfu.edu.cn

Qiang Weng, Ph.D., Laboratory of Animal Physiology, College of Biological Sciences and Technology, Beijing Forestry University, Beijing 100083, China. Fax: +86 10-6233-6399; E-mail: qiangweng@bjfu.edu.cn

Supplementary Figure S1

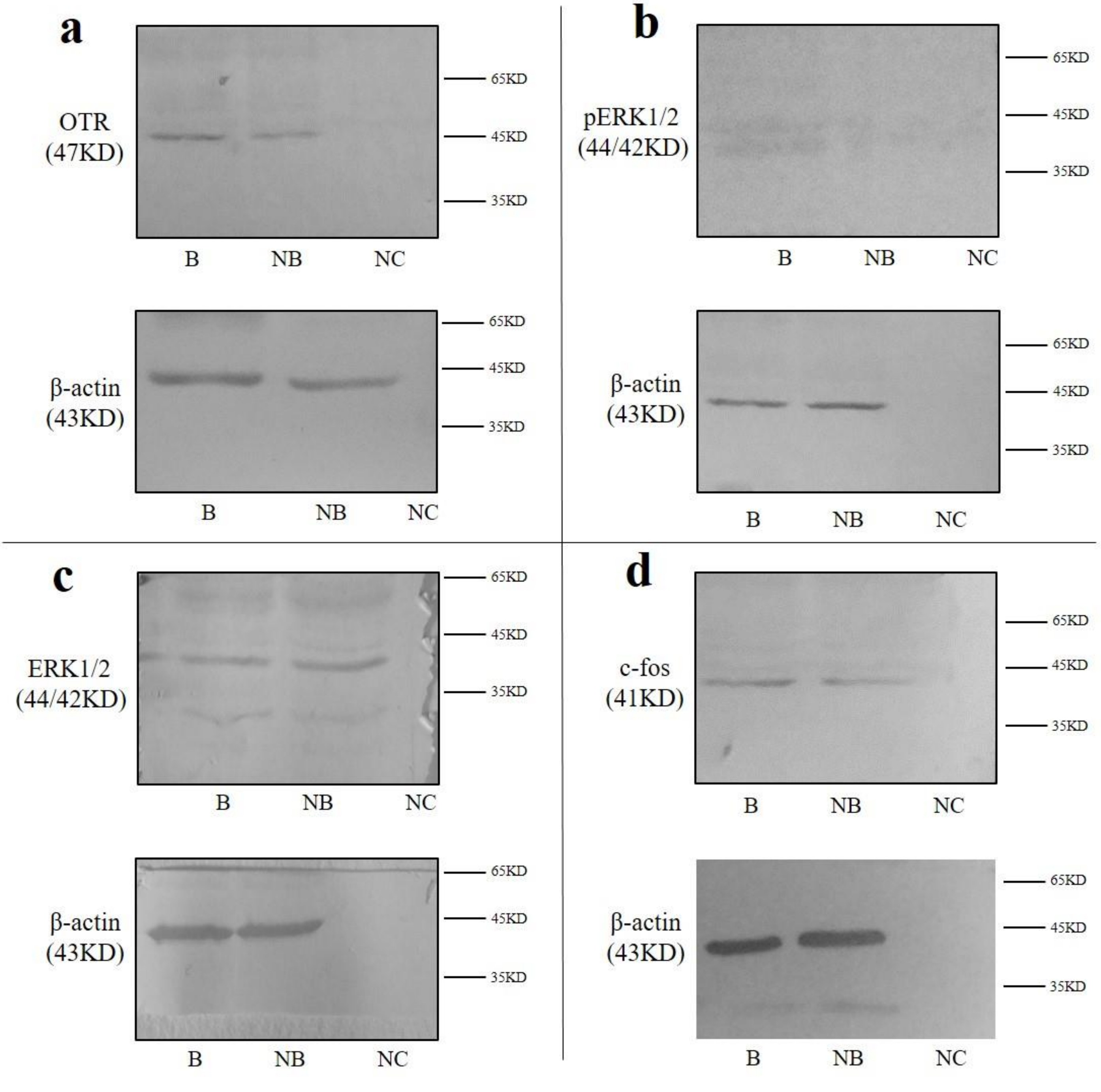

Figure S1: Full-length blots of Figure 4. (**a**) corresponds to Figure 4a; (**b**) corresponds to Figure 4b; (**c**) corresponds to Figure 4c; (**d**) corresponds to Figure 4d. B, breeding season; NB, non-breeding season; NC, negative control.

Supplementary Figure S2

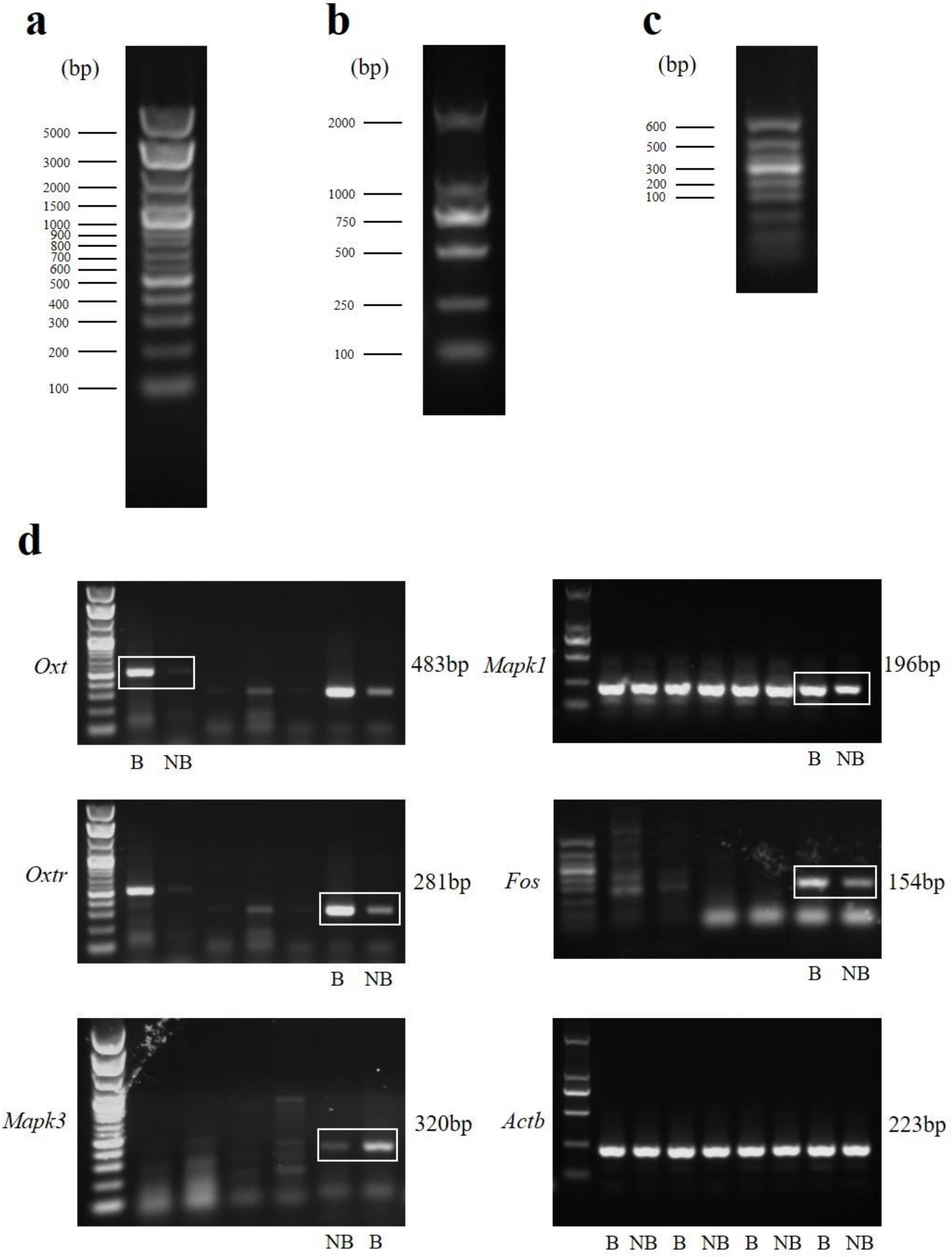

Figure S2: (**a-c**) Three different DNA markers. (**a**) corresponds to *Oxt*, *Oxtr* and *Mapk3*; (**b**) corresponds to *Mapk1* and *Actb*; (**c**) corresponds to *Fos*. (**d**) Original agarose gels of *Oxt*, *Oxtr*, *Mapk3*, *Mapk1*, *Fos* and *Actb* mRNA, respectively. White rectangles indicate the lanes assembled in the Figure 5. B, breeding season; NB, non-breeding season.
